# Accreditation System and Standards for Medical Education in Pakistan: It’s time we raise the bar

**DOI:** 10.12669/pjms.336.14178

**Published:** 2017

**Authors:** Ahsan Sethi, Arshad Javaid

**Affiliations:** 1 Dr. Ahsan Sethi, BDS (Pak), MPH (Pak), MMEd (Dundee, UK), FHEA (UK), MAcadMEd (UK), PhD Medical Education (Dundee, UK) Assistant Professor, Institute of Health Professions Education and Research, Khyber Medical University, Pakistan. ahsansethi@gmail.com; 2 Prof. Dr. Arshad Javaid, MBBS (Pak), MRCP (UK), MRCP (Ireland), MCPS (Pak), FRCP (UK), CCST (UK) Vice Chancellor, Khyber Medical University, Pakistan. arshadj34@gmail.com

There is a growing trend towards quality assurance and accreditation of medical schools worldwide.^1^ Globally, countries use accreditation and standards as regulatory mechanisms to ensure the quality of medical education, and in turn healthcare. Liaison Committee on Medical Education and Committee on Accreditation of Canadian Medical Schools set standards and accredit medical schools in the USA and Canada respectively.^2^ Ireland, Australia and many other countries have adapted World Federation for Medical Education (WFME) standards for accreditation purpose.^3^

WFME is an international body, with the goal to improve the quality of medical education globally.^4^ WFME published first set of standards for Basic Medical Education in 2003 and further refined them in 2012 and 2015,^1^, ^3^, ^4^ based on feedback from international experts. Their purpose is to encourage self-evaluation and bring improvements among institutions providing medical education.^3^, ^4^ WFME has already provided recognition status to seven accreditation agencies (from Japan, Caribbean, Korea, USA, Canada and Turkey) and several others (from Thailand, Sudan, Brazil, Mexico, Egypt etc.) are under review.^5^ WFME recognition, although not mandatory, helps in establishing global credibility of national accreditation agencies and strengthens collaboration among them.

WFME standards are structured under nine areas with 35 sub-areas, at two levels: ‘basic standards’ or minimum requirements; and relatively advanced standards called ‘quality standards’. Basic standards are mandatory while quality standards are optional. WFME claims that these standards are generic enough to be applied at global level, however, no study has been conducted in Pakistan to explore their appropriateness to Pakistani medical education context and how WFME standards and PMDC regulations can be merged to create a new set of standards for Pakistani medical and dental colleges.

The Educational Commission for Foreign Medical Graduates (ECFMG) assesses and certifies international medical graduates (IMGs) readiness for entry into US graduate medical education. ECFMG has announced that, after 2023, all applicants for certification will be required to have graduated from an accredited medical school.^2^ As the deadline is approaching, there is a need for accreditation bodies worldwide to get recognized by WFME, which has been entrusted by ECFMG for this purpose. One can argue that this requirement will only impact IMGs who wish to enter the US healthcare system, but it can be seen as a stimulus to harmonize accreditation standards and procedures for promoting excellence in medical education worldwide.

Pakistan Medical & Dental Council (PMDC) is the sole authority for accrediting, regulating and ensuring the quality of medical education in Pakistan. First set of PMDC regulations were developed in 1962 and the most recent ones were published in 2016.^6^ Here standards refer to the criteria, guidelines or characteristics aimed at achievement of desired quality levels. These standards (called ‘regulations’ by the PMDC), when imposed as a mandatory or legal requirement by the government take the form of regulations. Ironically, current PMDC accreditation process^6^ focuses predominantly on evaluation of infrastructure and ‘head counting’ in medical colleges, rather than measurement of educational processes and outcomes. The inspection process ensures that certain criteria (mostly emphasizing presence of infrastructure) are being met but it does not indicate how the presence of physical facilities and faculty translate into quality outcomes. This is high time that PMDC develops a new set of robust standards that are valid, reliable, acceptable, measurable and compatible with both, the local context and changing global scenario. In addition to developing new standards, PMDC also needs to review and redesign its accreditation system and strengthen its capacity to ensure that over 130 medical and dental colleges in Pakistan comply with new standards.

In response to the need for strengthening Pakistan accreditation system and standards, Institute of Health Professions Education and Research (IHPER) at Khyber Medical University (KMU) has designed a project: ‘Pakistan Accreditation System and Standards for Medical Education (PASS-ME)’. The project aims to review and redesign the current accreditation system at the PMDC and provide a new set of standards by integrating the existing ‘PMDC regulations’ with ‘WFME and other standards’. The project consists of several research studies on the current PMDC accreditation system, exploring the appropriateness of WFME standards to medical education in Pakistan and identifying avenues to merge existing PMDC regulations with WFME and other standards to create a new set of standards that address local needs of the community and are globally acceptable as well. Developing national consensus on new accreditation system and standards shall be another challenging task. Khyber Medical University recognizes that bringing reforms to the existing medical curricula in its constituent and affiliated medical colleges is an essential precursor to applying new standards. Curriculum reforms are already underway to make the existing curriculum student centered, well integrated and compatible with the community needs.^7^

PASS-ME is a multi-stakeholder project offering an opportunity to rethink our educational structure, processes and technical capacity for quality assurance of 130+ medical and dental colleges in Pakistan. The standards developed may aid WFME future revisions and the lessons learnt can help meet the needs of other countries. It will also pave way for national/international benchmarking. KMU seeks collaboration and technical assistance from medical educationists, Ministry of National Health Services, Regulation and Coordination, PMDC and international organizations to improve the quality of medical and dental education in Pakistan through successful implementation of PASS-ME project.
